# Chemomodulatory Effect of the Marine-Derived Metabolite “Terrein” on the Anticancer Properties of Gemcitabine in Colorectal Cancer Cells

**DOI:** 10.3390/md21050271

**Published:** 2023-04-26

**Authors:** Reham Khaled Abuhijjleh, Dalia Yousef Al Saeedy, Naglaa S. Ashmawy, Ahmed E. Gouda, Sameh S. Elhady, Ahmed Mohamed Al-Abd

**Affiliations:** 1 Department of Pharmaceutical Sciences, College of Pharmacy, Gulf Medical University, Ajman 4184, United Arab Emirates; 2020mdd01@mygmu.ac.ae (R.K.A.); 2020mdd03@mygmu.ac.ae (D.Y.A.S.); 2 Department of Pharmacognosy, Faculty of Pharmacy, Ain Shams University, Abbassia, Cairo 11591, Egypt; naglaa.saad@pharma.asu.edu.eg; 3 Research Institute for Medical and Health Sciences, University of Sharjah, Sharjah 27272, United Arab Emirates; 4 Life Science Unit, Biomedical Research Division, Nawah Scientific, Al-Mokkatam, Cairo 11571, Egypt; ahmed.gouda@nawah-scientific.com; 5 Department of Natural Products, Faculty of Pharmacy, King Abdulaziz University, Jeddah 21589, Saudi Arabia; ssahmed@kau.edu.sa; 6 National Research Centre, Department of Pharmacology, Medical and Clinical Research Institute, Cairo 12622, Egypt

**Keywords:** terrein, gemcitabine, combination analysis, colorectal cancer, cell cycle, apoptosis, autophagy, metabolomics, qPCR

## Abstract

Background: Terrein (Terr) is a bioactive marine secondary metabolite that possesses antiproliferative/cytotoxic properties by interrupting various molecular pathways. Gemcitabine (GCB) is an anticancer drug used to treat several types of tumors such as colorectal cancer; however, it suffers from tumor cell resistance, and therefore, treatment failure. Methods: The potential anticancer properties of terrein, its antiproliferative effects, and its chemomodulatory effects on GCB were assessed against various colorectal cancer cell lines (HCT-116, HT-29, and SW620) under normoxic and hypoxic (pO_2_ ≤ 1%) conditions. Further analysis via flow cytometry was carried out in addition to quantitative gene expression and ^1^HNMR metabolomic analysis. Results: In normoxia, the effect of the combination treatment (GCB + Terr) was synergistic in HCT-116 and SW620 cell lines. In HT-29, the effect was antagonistic when the cells were treated with (GCB + Terr) under both normoxic and hypoxic conditions. The combination treatment was found to induce apoptosis in HCT-116 and SW620. Metabolomic analysis revealed that the change in oxygen levels significantly affected extracellular amino acid metabolite profiling. Conclusions: Terrein influenced GCB’s anti-colorectal cancer properties which are reflected in different aspects such as cytotoxicity, cell cycle progression, apoptosis, autophagy, and intra-tumoral metabolism under normoxic and hypoxic conditions.

## 1. Introduction

Cancer is a major health problem worldwide, and the global burden of cancer has caused 10 million deaths in the past year [1]. While recent advances in the development of antitumor agents have contributed to cancer therapy, resistance to chemotherapy has led to recurrence and relapse [2]. In addition, the use of current antitumor agents has been limited due to their toxic and deleterious effects [3]. This calls for the development of novel anticancer agents with high efficacy to combat these issues.

Solid tumors suffer from a harsh microenvironment that has unique features and characteristics such as having areas with compromised endothelium, poor, or avascularized areas hindering drugs from reaching their targets, hypoxia in certain areas within the solid tumor, and a high level of acidosis with a noticeable pH gradient along the tumor tissue [4]. All the above features, nonetheless hypoxia, can play a role as drug targets due to the challenges faced with traditional chemotherapies. Oxygen levels in addition to the availability of nutrients differ drastically during the development of tumor cells through angiogenesis, and the recruitment of leukocytes and fibroblasts. Therefore, hypoxia can affect gene expressions, signaling pathways, many metabolic reactions, and the response to stress as well as the response to cytotoxic drugs [5]. Anticancer agents target various survival/death mechanisms or pathways in cancer cells, including angiogenesis, cell cycle regulation, apoptosis, and autophagy [6,7,8,9]. 

The secondary marine metabolite (+)-terrein was first isolated from *Aspergillus terreus* in 1935 and subsequently drew significant attention due to its various bioactivities, including anticancer properties [10]. Promising studies showed that terrein inhibits angiogenin production and secretion [11,12], induces cell cycle arrest [13,14] and apoptosis [15], and inhibits cell proliferation [16]. There is also evidence that terrein has anti-inflammatory activity that is mediated via inactivating the nuclear factor kappa B (NF-kB) signaling pathway. This occurs because of various mechanisms, most notably the inhibition of p60/p50 heterodimer translocation into the nucleus and the DNA-binding activity of the p65 subunit [12,17]. The NF-kB pathway has been implicated in various types of cancer due to its role in regulating apoptosis [18], and its abnormal activation can lead to malignant tumors and oncogenesis [19]. Yet, one of the major obstacles to using terrein on a large scale is the very low yield of this compound from different marine sources [20]. In addition, terrein and other marine-derived secondary metabolites are known for their abundance in diverse geographical distribution and their unique chemical structure [21].

Gemcitabine is the standard drug of choice for locally advanced and metastatic pancreatic cancer [22]. However, it is frequently associated with treatment failure due to intrinsic or acquired resistance. The failure of achieving good clinical outcomes in terms of survival could partially be associated with the hypo-vascularized and dense tumor stroma, and therefore, poor drug penetration and hypoxia [23]. Most patients acquire resistance after weeks of treatment, resulting in poor survival. Gemcitabine resistance can be either intrinsic or acquired and can result from molecular and cellular changes, such as nucleotide metabolism, apoptosis pathway suppression [24], ABC transporter protein overactivation/over-expression [25], activation of the cancer stem cells CSCs [26], activation of the epithelial-to-mesenchymal transition pathway (EMT) [27], and extracellular signal-regulated protein kinase (ERK) 1/2 overactivity [28]. Gemcitabine resistance is also associated with multiple genetic and epigenetic abnormalities. Changes in one or a few genes remain crucial for maintaining drug resistance, cell survival, and malignant phenotype [22]. There is evidence to indicate that NF-κB [29,30,31,32], AKT [29,33,34], MAPK [34,35], and HIF-1α pathways [36] are directly related to the resistance of gemcitabine in vitro and in vivo models.

Metabolomics is considered one of the best approaches to studying the effectiveness of drugs towards cancer [37] as well as assessing the reasons behind developing resistance toward drugs [38]. In this study, a metabolomic analysis has been conducted to compare the effect of terrein, gemcitabine, and a combination of both drugs on colorectal cells under normoxic as well as hypoxic conditions. ^1^HNMR spectroscopy was utilized in combination with chemometric statistical methods to compare the extracellular metabolites after each treatment.

Herein, we evaluated the interaction between terrein and gemcitabine in colorectal cancer cells under normal and hypoxic conditions in terms of a potential chemomodulatory effect and mutual intra-tumoral metabolic influence. 

## 2. Results

### 2.1. Cytotoxicity Assessment

To study the effect of Terr on the cytotoxic profile of GCB in colorectal cancer cell lines (HCT-116, HT-29, and SW620) under normoxic and hypoxic conditions, the viability dose–response curves of both agents, alone and in combination, were assessed using the E_max_ model as described in the Materials and Methods section. The IC_50_s of either agent, alone or in combination with their CI indices, are summarized in Table 1.

In the HCT-116 cells, GCB exerted potent cytotoxic activity despite a resistant fraction of 38.14 ± 1.4% in the normoxia condition; viability started to drop significantly (*p* < 0.05) from the control value at 0.03 μM and 0.01 μM in normoxia and hypoxia, respectively. The cellular log kill was gradual in profile with IC_50_ of 0.19 ± 0.028 μM and 0.01 ± 0.002 μM in normoxia and hypoxia, respectively. Terr exerted abrupt cytotoxic activity with increasing concentration; viability started to drop significantly at 100 μM in both conditions. The cellular log kill showed IC_50_ of (75.22 ± 0.97 μM and 20.26 ± 2.89 μM) in normoxia and hypoxia, respectively. An equitoxic combination of Terr with GCB improved the cytotoxic profile of GCB in the HCT-116 cell line in normoxia, decreasing the resistant fraction to 24.40 ± 1.22%; however, it did not improve the cytotoxic profile of GCB in hypoxia with an increased resistant fraction to 41.02 ± 2.33%. More so, the IC_50_ of GCB after combination with Terr decreased significantly compared to the single GCB treatment in normoxia and increased in hypoxia (Supplementary Figure S1A,B). The calculated CI values for GCB with Terr were 0.129 and 1.779 in normoxia and hypoxia, respectively. These CI values are indicative of synergistic interaction characteristics in the HCT-116 cell line under normoxic conditions and an antagonistic interaction under hypoxic conditions (Table 1).

For the HT-29 cells, GCB had a resistant fraction of 19.87 ± 12.0% and 42.64 ± 2.10% in normoxia and hypoxia, respectively; viability dropped significantly (*p* < 0.05) compared to the control cells at 0.01 μM and 0.03μM in normoxia and hypoxia, respectively (Supplementary Figure S2A,B). The IC_50_s of GCB were 0.01 ± 0.006 μM and 0.04 ± 0.004 μM in normoxia and hypoxia, respectively. The viability of the cells treated with Terr started to drop significantly (*p* < 0.05) from the concentration of 30 μM in normoxia and 100 μM in hypoxia. The IC_50_s of Terr were 56.24 ± 11.39 μM and 83.30 ± 4.41 μM in normoxia and hypoxia, respectively. An equitoxic combination of Terr with GCB did not improve the cytotoxic profile of GCB; however, it increased the resistant fractions to 46.45 ± 2.76% and 66.74 ± 6.84% in normoxia and hypoxia, respectively. The IC_50_ of GCB after the combination with Terr significantly increased compared to the single GCB treatment in normoxia and hypoxia. Yet, the calculated CI values for GCB with Terr were 2.318 and 7.277 in normoxia and hypoxia, respectively. These CI values are indicative of antagonistic interaction characteristics in the HT-29 cell line under both oxygen conditions.

With respect to SW620 cells, the resistant fraction after treatment with GCB was 32.62 ± 1.78% and 32.59 ± 0.28% in normoxia and hypoxia, respectively; viability dropped significantly (*p* < 0.05) at 0.3 μM in both normoxia and hypoxia. The IC_50_ of GCB was 0.21 ± 0.0003 μM, and 0.20 ± 0.008 μM in normoxia and hypoxia, respectively. The viability of the cells treated with Terr dropped significantly (*p* < 0.05) from the control value at 100 μM in both normoxia and hypoxia. The IC_50_ of Terr was 72.28 ± 1.35 μM, and 59.82 ± 8.50 μM in normoxia and hypoxia, respectively. An equitoxic combination of Terr with GCB improved the cytotoxic profile of GCB, keeping the resistant fraction at 32.30 ± 3.56% and 48.51 ± 2.97% in normoxia and hypoxia, respectively. IC_50_ of GCB after combination with Terr decreased compared to single GCB treatment in normoxia and hypoxia (Supplementary Figure S3A,B). The calculated CI values for GCB with Terr were 0.092 and 0.142 in normoxia and hypoxia, respectively. These CI values are an indicator of synergistic interaction characteristics in the SW620 cell line under both oxygen conditions.

### 2.2. The Influence of Terr on GCB-Induced Apoptotic Cell Death in Colorectal Cell Lines (HCT-116, HT-29, and SW620)

**Figure 1:**
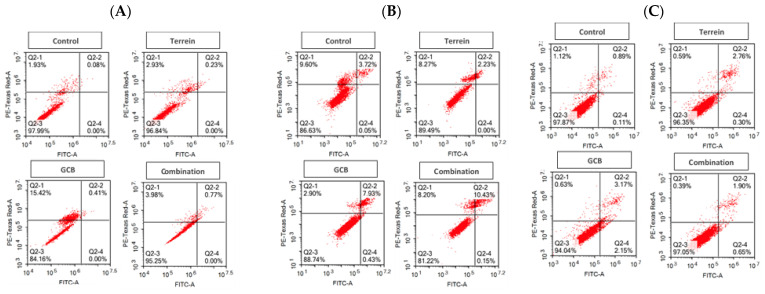

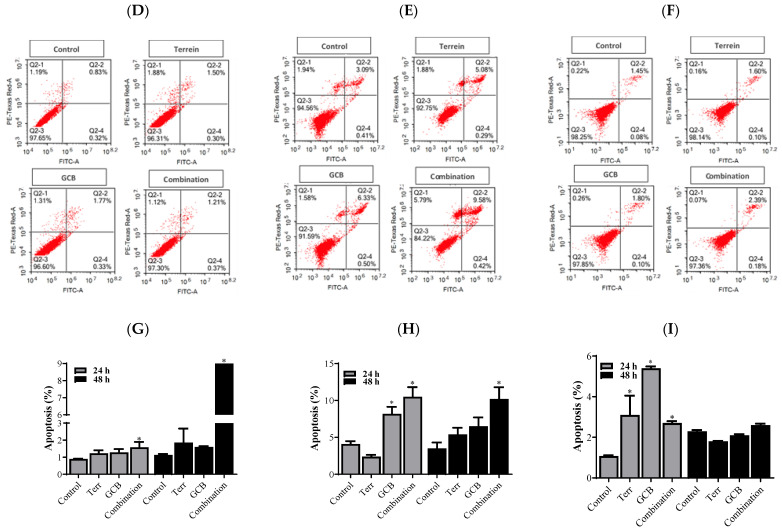
Programmed cell death (apoptosis) after treatment with Terr, GCB, and their combination for 24 h and 48 h. The cells were stained with annexin V-FITC/PI and different cell populations were plotted as a percentage of total events. HCT-116 under normoxia at 24 h and 48 h (**A**,**D**,**G**), HT-29 under normoxia at 24 h and 48 h (**B**,**E**,**H**), and SW620 under normoxia at 24 h and 48 h (**C**,**F**,**I**). Data are presented as mean ± SD; *n* = 3. * Significantly different from control group.

The tested colorectal cancer cells were exposed to the predetermined IC_50_ for 24 h and 48 h and stained with annexin V-FITC/PI. In HCT-116, only the combination treatment (GCB + Terr) significantly induced apoptosis after 24 h and 48 h of exposure (0.51 ± 0.22% and 9.59 ± 0.85%, respectively) compared to the control untreated cells (0.133 ± 0.05% and 3.99 ± 0.37%, respectively) (Figure 1A,D). Single treatments showed no significant difference in terms of apoptosis compared to the control untreated cells. Alternatively, GCB alone induced significant necrosis after 24 h of exposure (14.77 ± 1.06%) compared to the control untreated cells.

In the HT-29 cells, the GCB treatment significantly induced apoptosis after 24 h and 48 h of exposure (8.29 ± 0.92% and 6.06 ± 0.069%, respectively) compared to the control untreated cells (1.24 ± 0.21% and 1.26 ± 0.11%, respectively). The combination treatment (GCB + Terr) was found to significantly decrease apoptosis after 24 h and 48 h of exposure when compared to GCB alone (10.5 ± 0.45% and 10.2 ± 0.09%, respectively). On the other hand, there was no significant difference between GCB and Terr in terms of apoptosis at both time points (Figure 1B,E,H). 

In the SW620 cells, treatment with GCB significantly induced apoptosis after 24 h and 48 h of exposure (6.84 ± 0.47% and 2.1 ± 0.13%, respectively) compared to the control untreated cells (1.61 ± 0.28% and 2.24 ± 0.01%, respectively) (Figure 1C,F,I). The combination treatment (GCB + Terr) also increased apoptosis significantly after 24 h and 48 h of exposure (2.97 ± 0.91% and 2.84 ± 0.16%, respectively) when compared to the control untreated cells or GCB treatment alone. The combination treatment (GCB + Terr) induced significant necrosis after 24 h of exposure compared to the control untreated cells (5.17 ± 0.53% and 1.77 ± 0.17%, respectively). This effect was carried forward and influenced the total cell death, where the combination treatment (GCB + Terr) showed a significant increase in total cell death (14.14 ± 1.4%) compared to the control untreated cells (5.38 ± 0.19%) as well as single treatments (GCB or Terr).

**Figure 2:**
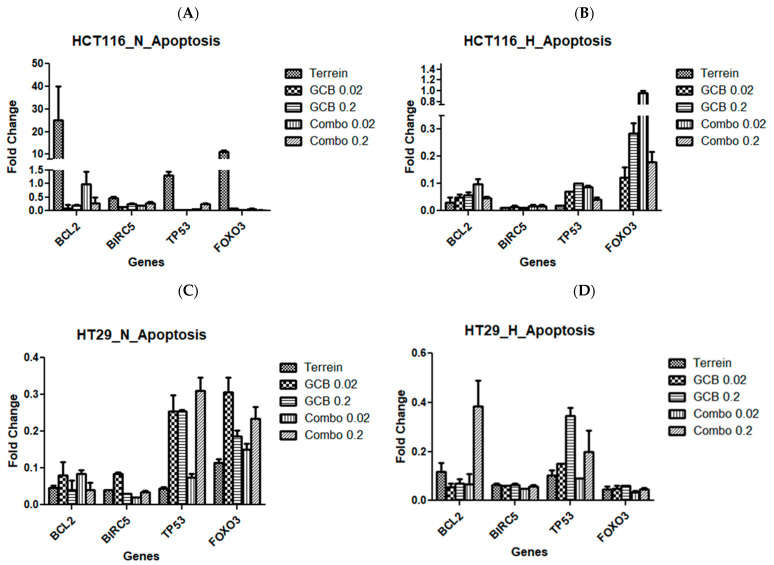

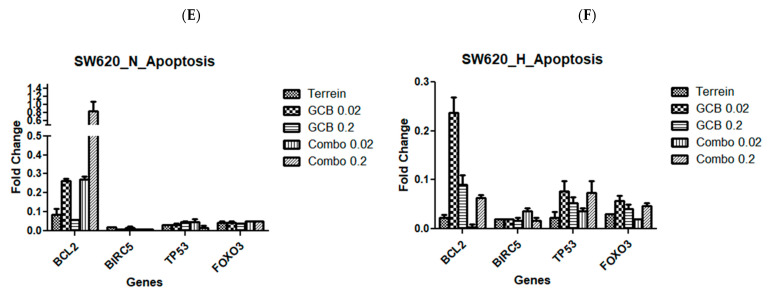
Fold change of apoptosis regulator genes after treatment with Terr, GCB, and their combination under normoxic and hypoxic conditions. A fold change value below 0.5 indicates under-expression, above 2.0 indicates over-expression, and between 0.5 and 2.0 indicates no change in expression. HCT-116 at 24 h under normoxia and hypoxia (**A**,**B**), HT-29 at 24 h under normoxia and hypoxia (**C**,**D**), and SW620 at 24 h under normoxia and hypoxia (**E**,**F**). The data are presented as mean fold change ± SD.

To further confirm the flowcytometric apoptosis-driven results, we examined apoptotic regulator genes using the RT-qPCR technique and calculated their fold changes after treatment under normoxic and hypoxic conditions. In HCT-116, the antiapoptotic gene, BCL2, was over-expressed by 25-fold when the cells were treated with terrein alone under normoxic conditions. The same gene showed no significant change in expression after the combination treatment (GCB + Terr). The rest of the treatments in both normoxic and hypoxic conditions resulted in the under-expression of BCL2. Similarly, the apoptosis inhibitor gene, BIRC5, was under-expressed in all treatments in both oxygen conditions. On the other hand, the expression of the tumor suppressor gene, TP53, was not affected after treatment with terrein in normoxia but was under-expressed in all other treatments in both conditions. Similarly, the apoptotic FOXO3 gene showed significant over-expression by 11-fold in normoxia when the cells were treated with terrein. FOXO3 showed no change in expression when the cells were treated with the combination treatment (GCB + Terr) under hypoxic conditions. The rest of the treatments in both conditions resulted in the under-expression of FOXO3 (Figure 2A,B). In HT-29, all genes tested for apoptosis were under-expressed for all treatments under both normoxia and hypoxia (Figure 2C,D). Similarly, in the SW620 cells, all genes that were tested for apoptosis were under-expressed with all treatments under both normoxia and hypoxia (Figure 2E,F).

**Figure 3:**
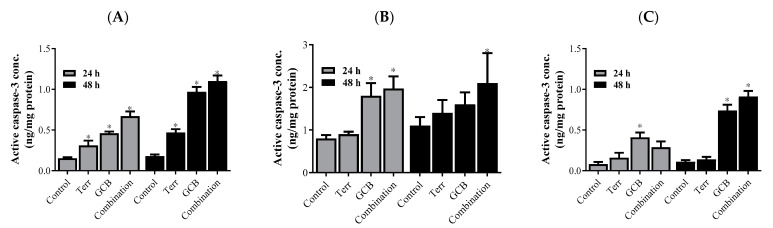
Caspase-3 level. The active caspase-3 concentration after treatment with Terr, GCB, and their combination in HCT-116 cells (**A**), HT-29 cells (**B**), and SW-620 cells (**C**) for 24 h. The data are presented as mean fold change ± SD. * Significantly different from control untreated cells.

Caspase-3 is crucial in the apoptosis process and is considered the executioner active caspase family member; its concentration indicates the actual progression of apoptosis. Herein, active caspase-3 was increased in HCT-116 in response to all single and combined treatments after 24 h and 48 h as well (Figure 3A). However, it was significantly increased in HT-29 when treated with the terrein and GCB combination for 24 and 48 h (Figure 3B). Similarly, the combination of terrein and GCB activated caspase-3 after 48 h only. Surprisingly, GCB induced the activation of caspase-3 after 24 h and 48 h as well (Figure 3C).

### 2.3. The Effect of Terr on the Autophagic Cell Death of Colorectal Cell Lines Treated with GCB

**Figure 4:**
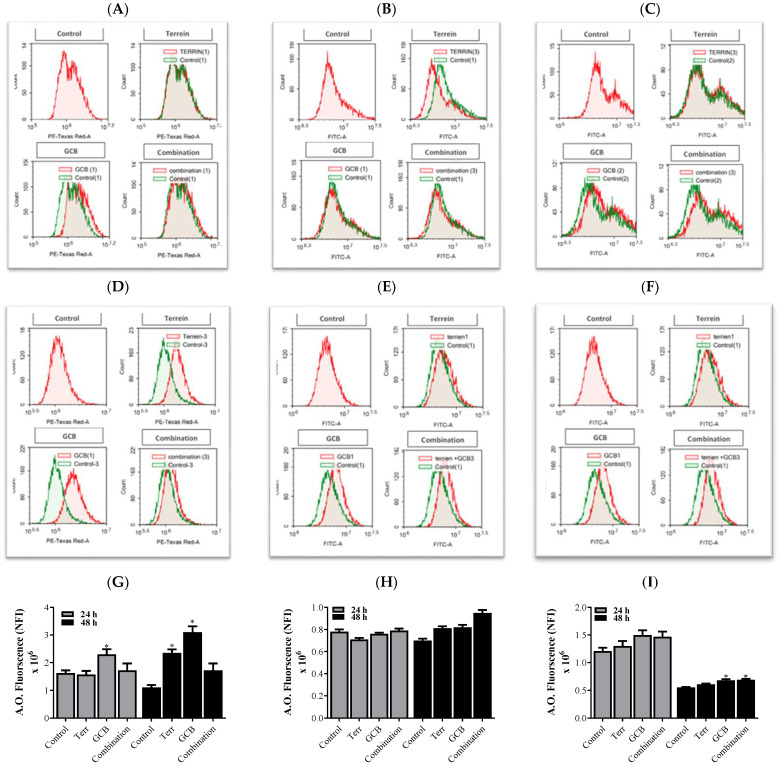
Induction of programmed cell death (autophagy) by Terr, GCB, and their combination for 24 h and 48 h. The cells were stained with acridine orange. The Average Net Fluorescent Intensity (NFI) values were plotted and compared to control cells. HCT-116 after 24 h and 48 h (**A**,**D**,**G**), HT-29 after 24 h and 48 h (**B**,**E**,**H**), and SW620 after 24 h and 48 h (**C**,**F**,**I**). Data are presented as mean ± SD; *n* = 3. * Significantly different from control untreated cells.

In the HCT-116 cells, GCB induced a significant increase in autophagic cell death by 54.63% compared to the control untreated cells after 24 h under normoxic conditions. Surprisingly, not only Terr but also the combination of GCB + Terr had no significant autophagic effect (Figure 4A,D,G).

In the HT-29 cells, only a combination of GCB + Terr for 48 h induced significant autophagic cell death by 38.18% increase in acridine orange-fluorescent signal compared to the control untreated cells (Figure 4B,D,H).

In the SW620 cells, after 24 h of exposure to the treatment, GCB induced a significant increase in autophagic cell death by 87.62% when compared to the control untreated cells. There was no significant difference between GCB and Terr as single treatments. However, combination treatment (GCB + Terr) induced a significant decrease in autophagic cell death when compared to GCB alone by 35.58%. After 48 h of treatment, the combination treatment induced a significant increase in autophagic cell death by 36.79% when compared to the control untreated cells. However, the combination treatment induced no significant difference when compared with GCB alone (Figure 4C,F,I).

**Figure 5:**
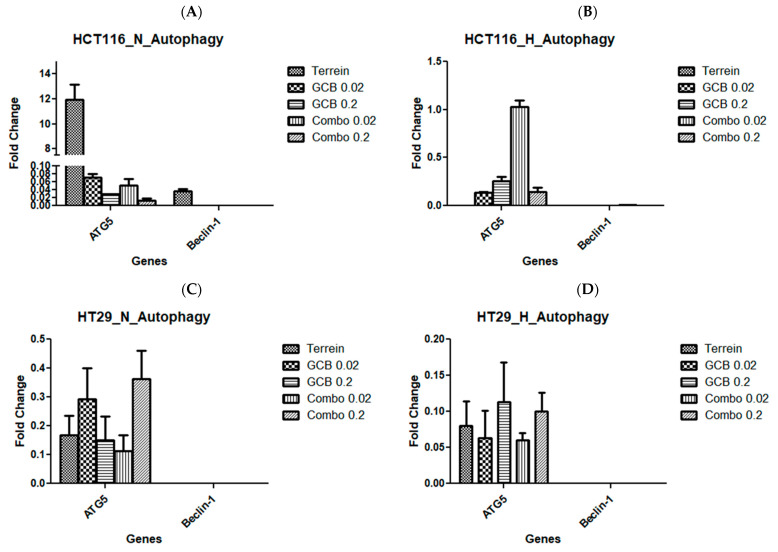

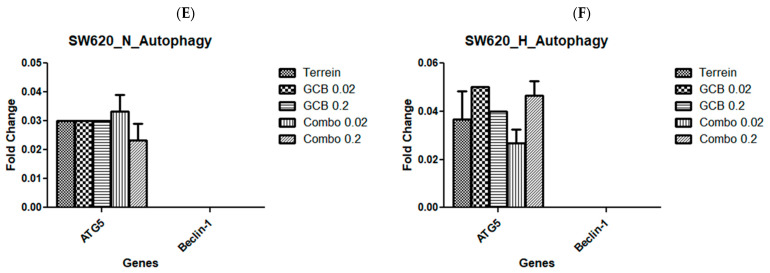
Fold change of autophagy regulator genes by Terr, GCB, and their combination under normoxic and hypoxic conditions. A fold change value below 0.5 indicates under-expression, above 2.0 indicates over-expression, and between 0.5 and 2.0 indicates no change in expression. HCT-116 cells under normoxia and hypoxia (**A**,**B**), HT-29 cells under normoxia and hypoxia (**C**,**D**), and SW620 cells under normoxia and hypoxia (**E**,**F**). The data are presented as mean ± SD.

To further confirm flowcytometric-driven results, we examined autophagy regulator genes (ATG5 and Beclin-1) using the RT-qPCR technique and calculated their fold changes after treatment under normoxic and hypoxic conditions. The autophagic-forming vesicle regulator gene, ATG5, was over-expressed when HCT-116 cells were treated with terrein under normoxic conditions by 12-fold. However, it was significantly under-expressed with the other treatments in both oxygen and hypoxia conditions. The golden autophagy standard gene, Beclin-1, was under-expressed in response to all treatments in both oxygen and hypoxia conditions (Figure 5A,B). In HT-29 and SW620, both genes tested for autophagy were under-expressed for all treatments under both normoxia and hypoxia (Figure 5C–F).

### 2.4. The Effect of Terr, GCB, and Their Combination on the Cell Cycle Distribution of Colorectal Cell Lines

**Figure 6:**
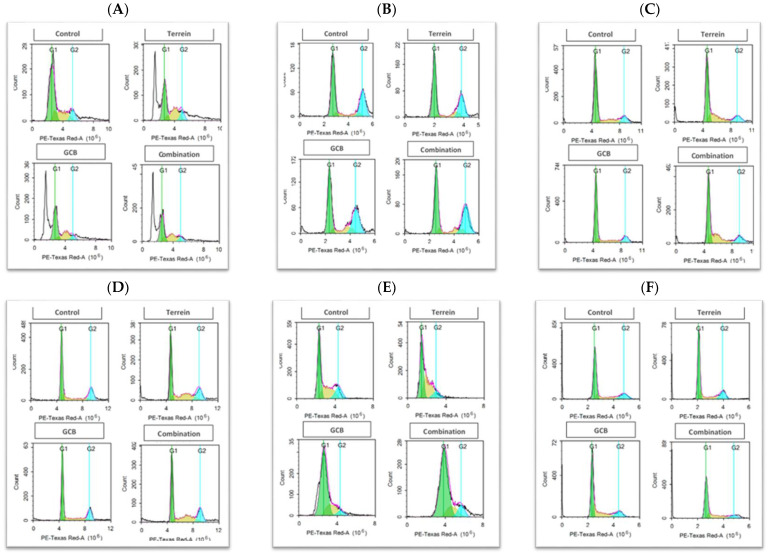
Effect of Terr, GCB, and their combination on the cell cycle distribution after 24 h and 48 h. The cell cycle distribution was determined using DNA cytometry analysis and different cell phases were plotted as the percentage of total events. HCT-116 after 24 h and 48 h (**A**,**B**), HT-29 after 24 h and 48 h (**C**,**D**), and SW620 after 24 h and 48 h (**E**,**F**).

In the HCT-116 cells, Terr induced a significant G_2_/M phase arrest and increased the cell population after 24 h from 27.8 ± 1.1% to 38 ± 1.15%. This effect was reversed by GCB where the cell population in the S phase dropped significantly to 32.61 ± 0.5%. As a result, there was no significant difference between the combination treatment (GCB + Terr) and GCB and Terr alone. On the other hand, after 48 h of exposure, there was no significant difference observed between all treatments in terms of the S phase population (Figure 6A,B).

In the HT-29 cells, Terr did not induce significant changes at the G_0_/G_1_ phase after 24 h of exposure. However, after further exposure (48 h), Terr induced a significant reduction in the cell population at the G_0_/G_1_ phase compared to the control untreated cells from 54.81 ± 0.7% to 42.78 ± 1.61%. Terr induced significant G_2_/M phase arrest after 24 h (from 22.84 ± 0.57% to 26.5 ± 1.99%) and after 48 h (from 22.42 ± 0.99% to 34.42 ± 0.84%). After 24 h of exposure, the combination treatment (GCB + Terr) induced a decrease in the G_0_/G_1_cell population when compared to GCB alone from 67.3 ± 1.81% to 54.32 ± 1.49%, where the combination treatment (GCB + Terr) increased the S phase cell population compared to GCB (from 18.48 ± 0.09% to 31.39 ± 0.92%). Similar results were observed after 48 h of exposure (Figure 6C,D).

In the SW620 cells, GCB increases in the G_0_/G_1_ phase cell population after 24 h of exposure from 48.04 ± 1.28% to 77.43 ± 2.57%. This effect was also seen for the combination treatment (GCB + Terr), where the cell population in the G_0_/G_1_ phase increased significantly to 52.77% when compared to the control untreated cells. After 48 h of exposure, the combination treatment induced significant G_2_/M phase arrest and increased the S phase cell population from 18.96 ± 0.86% to 27.88 ± 1.14% when compared to the control untreated cells. It also increased the G_0_/G_1_ phase cell population from 53.01 ± 2.72% to 60.16 ± 1.08% compared to GCB alone (Figure 6E,F).

**Figure 7:**
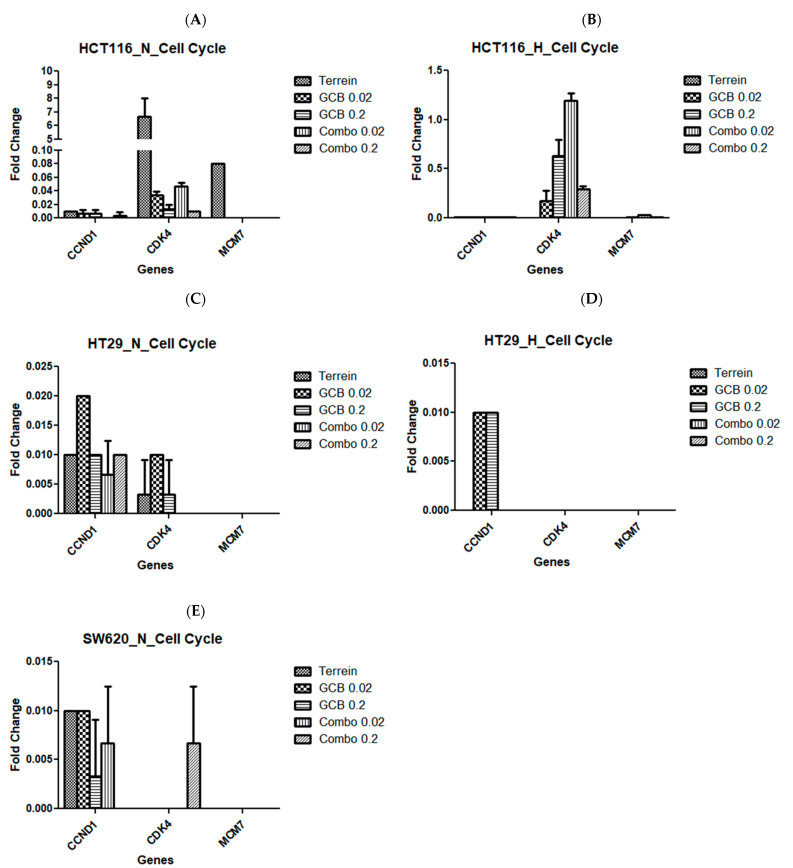
Fold change of cell cycle regulatory genes due to treatment with Terr, GCB, and their combination under normoxic and hypoxic conditions. A fold change value below 0.5 indicates under-expression, above 2.0 indicates over-expression, and between 0.5 and 2.0 indicates no change in expression. HCT-116 under normoxia and hypoxia (**A**,**B**), HT-29 under normoxia and hypoxia (**C**,**D**), and SW620 under normoxia and hypoxia (**E**). The data are presented as mean fold change ± SD.

In addition, we examined cell cycle regulatory genes by RT-qPCR (CCND1, CDK4, and MCM7). In HCT-116, only CDK4 was over-expressed due to treatment with terrein by 6.7-fold under normoxic conditions. CCND1 was under-expressed in response to all treatments under both oxygen and hypoxia conditions. Similar results were observed with the MCM7 gene (Figure 7A,B). In HT-29 and SW620, all genes tested for cell cycle regulation were under-expressed in response to all treatments under both normoxia and hypoxia (Figure 7C–E).

### 2.5. The Effect of GCB, Terr, and Their Combination on the Colorectal Cell Lines’ Growth and Proliferation

The combination of Terr and GCB was found to show a synergistic effect in normoxia and an antagonistic effect in hypoxia. This was evident when checking the cell growth regulators and cell proliferation regulators in both normoxia and hypoxia conditions. We examined and quantified the fold changes of several genes responsible for cellular growth (AKT1, TGF-B1, HIF1-α, and PRKDC) and cellular proliferation (PCNA and RAD18) using the RT-qPCR technique.

**Figure 8:**
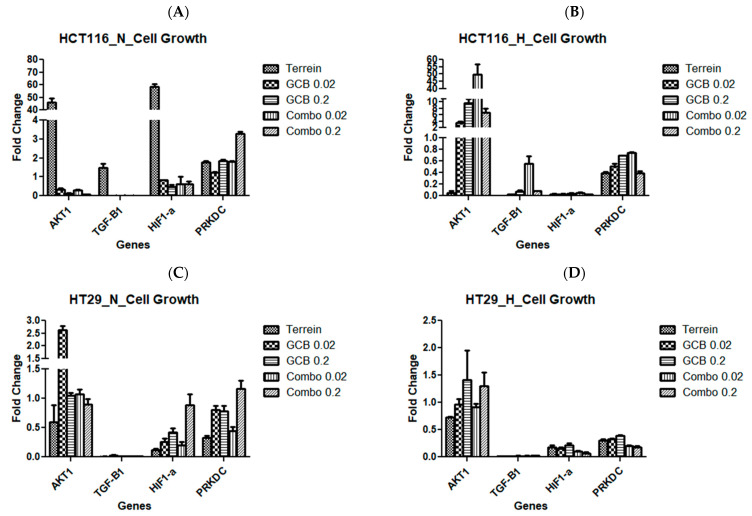

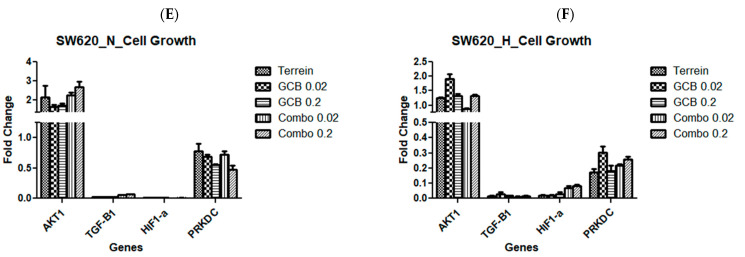
Fold change of genes by Terr, GCB, and their combination under normoxic and hypoxic conditions. A fold change value below 0.5 indicates under-expression, above 2.0 indicates over-expression, and between 0.5 and 2.0 indicates no change in expression. HCT-116 under normoxia and hypoxia (**A**,**B**), HT-29 under normoxia and hypoxia (**C**,**D**), and SW620 under normoxia and hypoxia (**E**,**F**). The data are presented as mean fold change ± SD.

**Figure 9:**
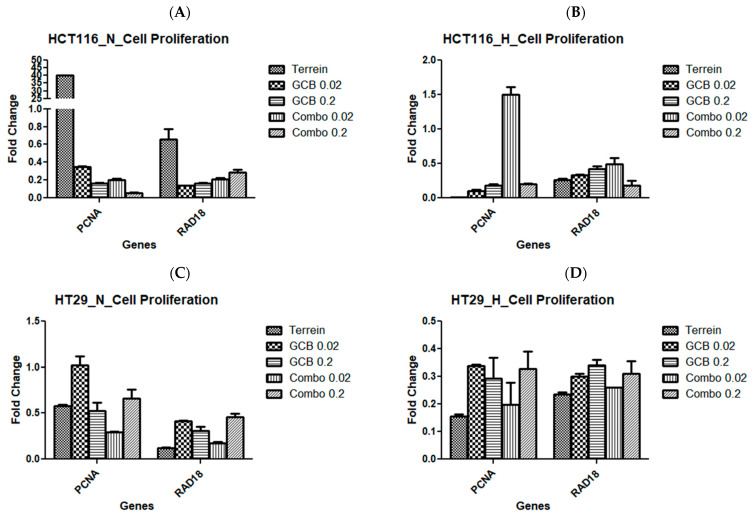

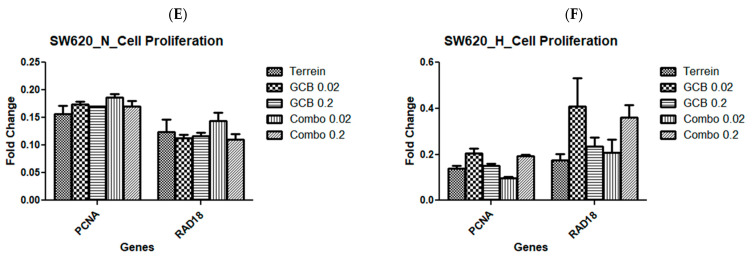
Fold change of genes by Terr, GCB, and their combination under normoxic and hypoxic conditions. A fold change value below 0.5 indicates under-expression, above 2.0 indicates over-expression, and between 0.5 and 2.0 indicates no change in expression. HCT-116 at 24 h under normoxia and hypoxia (**A**,**B**), HT-29 at 24 h under normoxia and hypoxia (**C**,**D**), and SW620 at 24 h under normoxia and hypoxia (**E**,**F**). The data are presented as mean fold change ± SD.

In HCT-116, the AKT1 gene was over-expressed when the cells were treated with terrein in normoxia by 45.8-fold and were under-expressed for other treatments in the same condition. The contrary was evident in hypoxia. TGF-1β showed no change in expression for cells treated with terrein but was under-expressed when the cells were treated with other treatment conditions under normoxia. HIF1-α showed similar results to the ones seen by AKT1 under normoxic conditions. PRKDC showed no change in expression for all treatments except the combination treatment (GCB + Terr) under normoxic conditions. At hypoxia, TGF-1β, HIF1-α, and PRKDC were under-expressed in all treatments (Figure 8A,B and Figure 9A,B).

On the other hand, the cellular proliferation gene, PCNA, was over-expressed when the cells were treated with terrein in normoxic conditions by 40-fold. However, it was under-expressed for the rest of the treatments. At hypoxia, the combination treatment (GCB + Terr), resulted in no change in the expression of PCNA. Yet, the remaining treatments resulted in the under-expression of the gene. The RAD18 gene was under-expressed in all treatments in both oxygen conditions except for terrein in normoxia where the gene did not change in expression (Figure 8A,B).

In HT-29, AKT1 was only over-expressed 2.62-fold after treatment with GCB. TGF-1β was under-expressed in all treatments under both oxygenation conditions. HIF1-α was under-expressed in all treatments and oxygen conditions, except after treatment with (GCB + Terr) where it showed no change in expression. PRKDC showed no change in expression in normoxia in response to all treatment conditions (Figure 8C,D). PCNA was under-expressed due to hypoxic conditions and no change in expression was observed under normoxia (Figure 9C,D).

In SW620, AKT1 showed no change in gene expression for all treatments under normoxia and hypoxia. However, TGF-1β and HIF1-α were under-expressed in all treatments under normoxia and hypoxia. The PRKDC gene was only expressed under hypoxia conditions for all treatments (Figure 8C,D). PCNA and RAD18 were under-expressed in this cell line for all treatment groups under both oxygenation conditions (Figure 9E,F).

### 2.6. The Effect of Terr, GCB, and Their Combination on the Extracellular Metabolites within Colorectal Cell Llines

^1^H-NMR comparative analysis was carried out between the metabolites released from HCT-116 cells after three different drug treatments: Terr only, GCB only, and a combination of both Terr and GCB under normoxic conditions. Three metabolites, namely, ethyl malonate, tyrosine, and methylhistidine were detected extracellularly from HCT-116 cells treated with GCB only. While other metabolites such as hypoxanthine and imidazole were identified in the HCT-116 cells treated with Terr and GCB + Terr but not in the extracellular fluid of the HCT-116 cells treated with GCB only. On the other hand, methionine was detected extracellularly in the HCT-116 cells treated with Terr only. Metabolites such as formate and pipocolate appeared extracellularly in both cell lines treated with Terr and GCB only but were not detected in cell lines treated with (GCB + Terr).

Another similar comparative analysis was performed between the metabolites released from HCT-116 cells after the same three different drug treatments but under hypoxic conditions. Three metabolites, namely, 4-hydroxyphenyl acetate, leucine, and pyruvate were detected only in the extracellular fluid of HCT-116 cells treated with both drugs (GCB + Terr) and were not detected in the cells treated only with single drugs. Furthermore, methionine and phenylalanine were detected in all the treatments except the GCB-treated cell lines.

Then, a third ^1^H-NMR metabolomic analysis was conducted to compare the extracellular metabolites released by HCT-116 cells in both hypoxic and normoxic conditions. A total of 25 metabolites were detected from HCT-116 cells treated with the three drugs under normoxic and hypoxic conditions. In total, 19 of them were common between the two conditions (Table 2). Three metabolites, namely, 2-hydroxy valerate and 2-phosphoglycerate, were completely absent from all normoxic treatments and detected in all hypoxic ones, while pyruvate was exceptionally detected in the hypoxic cells treated with the combined drugs (GCB + Terr). On the other hand, methylhistidine was uniquely detected in the normoxic cells treated only with GCB drugs.

**Figure 10:**
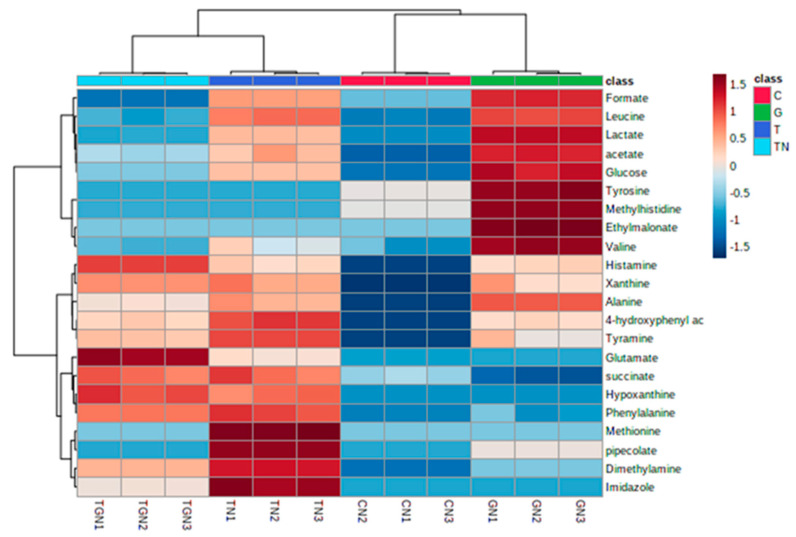
Unsupervised hierarchical clustering and heat map analysis of extracellular metabolites released from HCT-116 under normoxic conditions.

**Figure 11:**
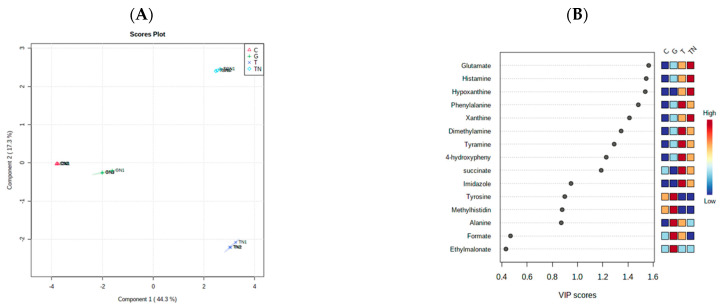
Supervised partial least squares discriminant analysis (PLS-DA) of the metabolite profiling of HCT-166 cells treated with Terr (T), gemcitabine (G), and Terr + GCB (TG) under normoxic conditions. (**A**) Two-dimensional score plot for HCT-116 cells treatments. (**B**) VIP score plot for treatments of HCT-116 cells.

Two multivariate statistical analyses, hierarchical cluster analysis (HCA) and partial least squares discriminant analysis (PLS-DA), were utilized to study the overall difference in the metabolites released because of the three different drug treatments under each of the normoxic and hypoxic conditions individually and then were thirdly conducted to compare between the two conditions. The first HCA classified the released metabolites under normoxic conditions after the three drug treatments into clusters based on their abundance (Figure 10). Furthermore, supervised multivariate analysis, PLS-DA, showed that metabolites from different treatments under normoxic conditions are separately clustered (Figure 11A). VIP scores were selected as a criterion for choosing the most important variables of the PLS-DA model (Figure 11B). For normoxic conditions, the significantly different metabolites with a VIP score of greater than 1 are glutamate, histamine, hypoxanthine, phenylalanine, xanthine, dimethylamine, tyramine, 4-hydroxyphenyl alanine, and succinate.

**Figure 12:**
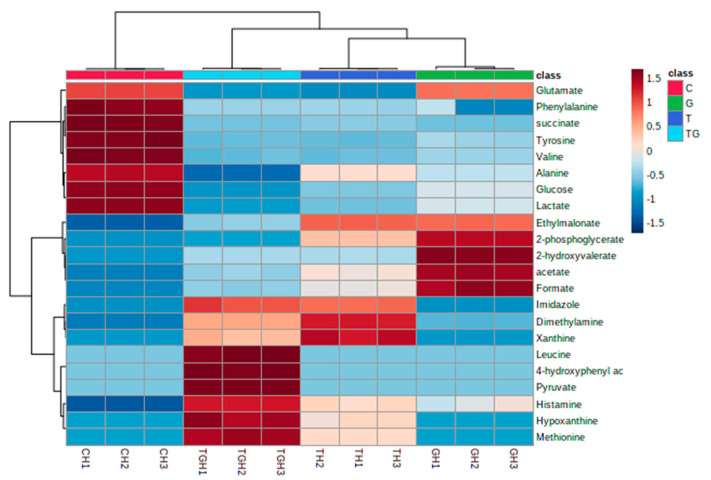
Unsupervised hierarchical clustering and heat map analysis of extracellular metabolites released from HCT-116 under hypoxic conditions.

**Figure 13:**
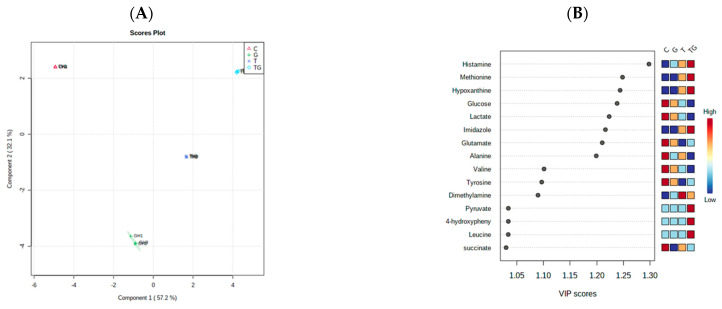
Supervised partial least squares discriminant analysis (PLS-DA) of the metabolite profiling of HCT-166 cells treated with Terr (T), gemcitabine (G), and Terr + GCB (TG) under hypoxic conditions. (**A**) Two-dimensional score plot for HCT-116 cells treatments. (**B**) VIP score plot for treatments of HCT-116 cells.

Similarly, hierarchical cluster analysis (HCA) and partial least squares discriminant analysis (PLS-DA) were conducted to investigate the difference in the metabolites released because of different kinds of drug treatments under hypoxic conditions. HCA clustered the released metabolites based on their abundance (Figure 12). PLS-DA revealed that metabolites from the three treatments are separately clustered (Figure 13A). VIP scores were also selected as criteria for choosing the most important variables of the PLS-DA model. The most significantly different metabolites with a VIP score of more than 1 as shown in (Figure 13B) are histamine, methionine, hypoxanthine, glucose, lactate, imidazole, glutamate, alanine, valine, tyrosine, dimethylamine, pyruvate, 4- hydroxyphenyl alanine, leucine, and succinate.

**Figure 14:**
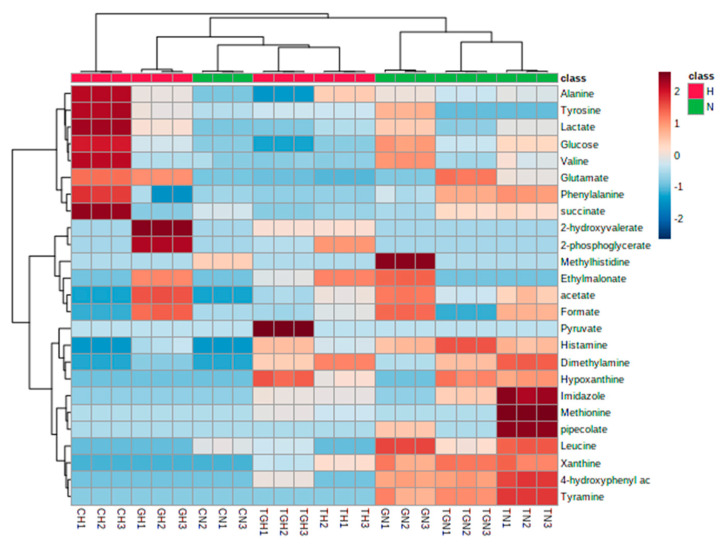
Unsupervised hierarchical clustering and heat map analysis of extracellular metabolites released from HCT-116 comparing normoxic and hypoxic conditions.

**Figure 15:**
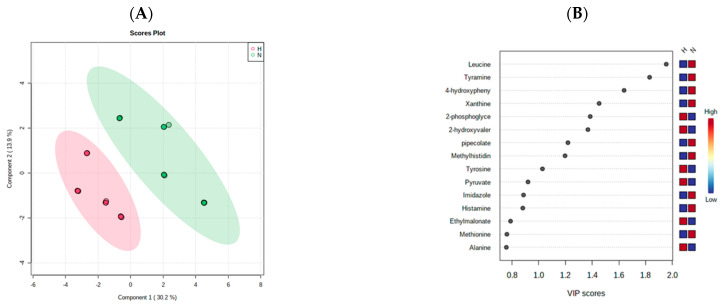
Supervised partial least squares discriminant analysis (PLS-DA) of the metabolite profiling of HCT-166 cells treated with Terr (T), gemcitabine(G), and Terr + GCB (TG) under normoxic and hypoxic conditions. (**A**) Two-dimensional score plot for HCT-116 cells treatments. (**B**) VIP score plot for treatments of HCT-116 cells.

The same multivariate analysis methods (hierarchical cluster analysis and partial least squares discriminant analysis) were applied to assess the difference in the metabolites released from HCT-116 cells under both normoxic and hypoxic conditions. The heat map showed the difference in metabolites’ concentration between hypoxic and normoxic treatments (Figure 14). Moreover, PLSD-A separated the hypoxic metabolites and normoxic metabolites into two clusters (Figure 15A). The most significantly different metabolites between hypoxic and normoxic cells were determined using VIP scores (Figure 15B).

## 3. Discussion

The need for novel anticancer treatment is an emerging matter as cancer is a major health problem worldwide, and the currently available chemotherapeutic options are becoming increasingly susceptible to resistance [1,2]. Terrein (Terr) is a bioactive marine metabolite isolated from the fungal strain of *Penicillium* species SF-7181 and *Aspergillus terreus* [39]. It exerts its activity via different mechanisms such as angiogenesis inhibition, cell cycle regulation, apoptosis, and autophagy induction [6,7,8,9]. However, the exact role of Terr as an antitumor agent remains unclear. Gemcitabine (GCB) is generally considered to be the drug of choice for pancreatic adenocarcinoma and has also been used in other types of cancers such as colorectal cancer; however, its major drawback is its susceptibility to both intrinsic and acquired chemoresistance [40]. Therefore, improving the therapeutic effect of GCB is crucial.

In the current work, Terr showed relatively high IC_50_s against three different colorectal cancer cell lines under normoxic and hypoxic conditions. Yet, several similar studies showed that Terr induces cancer cell death; however, it is highly cell type-dependent, as well as dose and time dependent [12,16]. On the other hand, GCB showed much higher anticancer potencies against the same set of cell lines compared to Terr under normoxic and hypoxic conditions. The combination indices for GCB with Terr were indicative of a synergistic interaction in HCT-116 and SW620; however, it was antagonistic in HT-29 in both normoxia and hypoxia.

To explain the characteristics of the interaction between GCB and Terr treatments as well as their combination, apoptosis, autophagy, and cell cycle interference were assessed using the flowcytometry technique. This allowed us to determine if the cell death was due to programmed/non-programmed (apoptosis vs necrosis) cell death, autophagy induction/suppression, or simply interference with cell cycle progression (antiproliferative properties). It is worth mentioning that the role of autophagy in cancer is very controversial as it is often referred to as either inducing cell death by suppressing tumorigenesis or facilitating tumorigenesis [41,42]. Apoptotic cell death in GCB singular treatment was significantly higher compared to control untreated cells in all cell lines assessed as expected. Yet, after 24 h of treatment in HCT-116 and SW620 cell lines, the combination treatment significantly increased apoptotic cell death compared to other treatments. The opposite was evident in the HT-29 cell line under both normoxic and hypoxic conditions. Both cases are in alignment with combination indices calculated in these cell lines (synergistic versus antagonistic). It is worth mentioning that apoptosis was induced, and autophagy was suppressed after 24 h in HCT-116 and SW620 cells under combination conditions (GCB + Terr). This might be attributed to suppressed autophagy leading to apoptosis and cell death. Furthermore, the gene expression profile of apoptosis and autophagy genes confirmed this pattern. In the previous literature, cells such as HCT-116 can proceed via apoptosis and suppress autophagy through the regulation of certain pathways, such as PI3K/AKT/mTOR [43]. It is prevalent in colorectal cancer cells and exhibits the antagonistic effect between autophagy and apoptosis as a survival mechanism due to crucial environmental factors [44]. Concerning cell cycle analysis, all cell lines that were tested showed a significant increase in the S phase population after 24 and 48 h of treatment with GCB, while Terr induced a significant increase in G_2_/M phase arrest in the same set of cell lines. Previous studies showed similar findings and have shown that GCB induces S-phase cell cycle arrest and regulates cell cycle-related proteins, while Terr induces G_2_/M phase cell cycle arrest [45,46].

RT-qPCR analysis for single and combined treatment was conducted for apoptosis-related genes (BCL2, BIRC5, TP53, and FOXO3), autophagy-related genes (ATG5 and Beclin-1), cell cycle-related genes (CCND1, CDK4, and MCM7), cellular growth-related genes (AKT1, TGF-B1, HIF1-a, and PRKDC) and cellular proliferation-related genes (PCNA and RAD18). Most of the apoptotic genes that were studied were under-expressed in all cell lines that were tested for all treatments under both normoxic and hypoxic conditions. The anti-apoptotic gene, BCL2, which plays a role in programmed cell death as an antiapoptotic protein [47], was over-expressed when HCT-116 cells were treated with Terr alone under normoxic conditions; however, it was significantly downregulated when the cells were treated with the combination treatment (GCB + Terr). This observation is supported by similar combination studies that showed the downregulation of BCL2 when HCT-116 was treated with combination treatment [48].

Similar results were observed with the autophagy-regulating genes; they were found to be under-expressed in all cell lines tested for all treatments under both normoxic and hypoxic conditions. ATG5, which regulates autophagy by forming autophagic vesicles and controls mitochondrial quality after oxidative damage, was over-expressed when HCT-116 cells were treated with Terr under normoxic conditions. According to the literature, Beclin-1 is a gene that plays a significant role in regulating autophagy, proliferation, and apoptosis in colorectal cancer cells (HCT-116 and SW620). The inhibition of Beclin-1 leads to the suppression of autophagy and proliferation as well as the promotion of apoptosis, which are observed in the results [49]. The exact mechanism by which Beclin-1 promotes apoptosis and suppresses autophagy, however, remains unclear.

Concerning cell cycle regulator genes, they followed the same pattern, and most of the genes that were tested were under-expressed in all cell lines that were tested for all treatments under both normoxic and hypoxic conditions. The only exception was CDK4, which is an important gene that encodes proteins for the cell cycle G1 phase progression. CDK4 was only over-expressed when the cells were treated with Terr under normoxic conditions in the HCT-116 cell line. However, the combination treatment (GCB + Terr) led to the downregulation of CDK4 in HCT-116. CDK4 is found to be amplified in colorectal cancer cells compared to normal cells [50,51], and evidence has shown that inhibiting certain CDKs such as CDK1, 2, 4/6, and 9 is useful in enhancing colorectal cancer cell (HCT-116) death [52]. According to previous studies, the inhibition of CDKs has also proven to be beneficial in suppressing colorectal cancer cells from proliferation through cell cycle arrests, and in some cases, can also lead to apoptotic cell death [53].

Most of the cellular growth genes that were tested were observed to be under-expressed in all cell lines in response to treatment. The AKT1 gene was of great interest and a known target for terrein, as it works by regulating various processes such as metabolism, proliferation, cell survival, and cell growth. In HCT-116, AKT1 was over-expressed when the cells were treated with Terr in normoxia and under-expressed for other treatments in the same condition. The contrary was evident in hypoxia. Hypoxia plays a major role in tumor cell behavior and the way it responds to treatment [54]. Stegeman et. al. showed that hypoxia stimulates AKT expression and activation in vivo and in vitro. The current study might prove that terrein-induced pAKT inhibition can overcome the influence of hypoxia and diminish cell survival in hypoxic cells, rather than in normoxic conditions [55]. In HT-29, AKT1 was over-expressed after treatment with GCB in normoxia, which might explain the antagonistic interaction with terrein. In SW620, AKT1 was over-expressed after single as well as combination treatment under both normoxic and hypoxic conditions. Still, this can explain the very high resistance fraction (R-value) to treatment in this cell line. AKT is known to regulate cellular proliferation through the degradation of CDK inhibitors, therefore promoting cell cycle progression and inhibiting apoptosis by inactivating pro-apoptotic molecules [56,57]. As a result, AKT plays a vital role as a signaling biomarker, which integrates many potential oncogenic signals [58]. On the other hand, recent studies showed that overactivation of AKT can also increase cell resistance to oxidative stress and allow cells to be more viable in high reactive oxygen species (ROS) conditions [59]. On the other hand, several studies showed that AKT nucleus translocation can induce cell death via apoptin due to the activity of some anticancer drugs [60]. This proves that the overall outcome of AKT activation or inhibition depends on the signaling context as well as the topological characteristics [58]. Most of the cell proliferation genes that were studied were under-expressed in response to single and combined treatment. Yet, this might indicate the antiproliferative effect rather than the cytotoxic properties of treatments under investigation, especially if we noticed the high resistance fraction in all treatment conditions.

Only HCT-116 cells showed a synergistic versus antagonistic interaction between terrein and GCB under normoxic versus hypoxic conditions, respectively. Yet, it was further assessed via a metabolomic study using ^1^H-NMR comparative analysis and profiling under normoxic versus hypoxic conditions. Lately, many studies have been trying to investigate colorectal cancer metabolic profiling compared to normal tissues [61]. Hirayama et al. found that due to hypoxia, there is a significant variation in the energy metabolism in the colorectal cancer tissues [62]. Denkert et al. reported that intermediates of the lipids and tricarboxylic acid (TCA) cycle were downregulated in tumor tissue, while urea cycle metabolites, purines, pyrimidines, and amino acids were upregulated compared to normal tissue [63]. In the current study, the extracellular metabolites of the different treatment conditions (single versus combination treatment) were found to be significantly different from one another when compared under different oxygen conditions. According to the VIP scores, the metabolites that were believed to exhibit a significant role in the metabolic shift when comparing the different oxygen conditions as well as when testing normoxia and hypoxia per se were leucine, tyramine, 4-hydroxyphenyl acetate, xanthine, and tyrosine. A study carried out by Hirayama et al. showed that tumors can have a tumor-specific metabolism that grants them more prevalent proliferation, at the same time keeping some metabolic characteristics of the tissues from which they originated. To put it in another way, cancer cells are progressed via metabolic adaptation that includes the upregulation of glucose consumption and increase in amino acids, while preserving the tissue-specific dependency of aerobic respiration characterized by TCA intermediate and nucleotide levels [62]. Another study performed by Frezza et al. suggests that hypoxic HCT116 cells could depend on catabolic processes to make up the energetic defect created by the loss of mitochondrial activity and that cannot be made up by the increased glycolytic flux [64]. Herein, these metabolic profiling data are in alignment with autophagy and apoptosis results. Mitochondrial membrane and integrity are among the early flip points in apoptosis/autophagy balance [44,65]. The significantly different profiles of energy metabolites in both normoxic and hypoxic conditions mirrored the different profiles in autophagy/apoptosis balance in the HCT-116 cells [66]. In our study, according to the PLS-DA, there was no overlap in metabolites clustering among the different treatments. Further analysis was conducted via a metabolite–gene–disease interaction network, which illustrated that some of the genes that were tested were directly linked to colorectal cancer in alignment with the metabolites that were found earlier, such as AKT1 and BCL2.

In conclusion, terrein possesses a controversial role in influencing the anticancer properties of gemcitabine in colorectal cancer cells under normoxic versus hypoxic conditions ranging from antagonism to synergism. However, this influence is evident via significant changes in cell proliferation patterns, cell cycle progression, apoptosis, and autophagy with mirrored confirmatory gene expression profiles. AKT1 seems crucial in all these processes in terms of activation and expression. On top of all these, the metabolic profile of energy and mitochondrial function was significantly and differentially affected by single and combined treatments under normoxia versus hypoxic conditions. It is recommended to further study these effects under more complicated tissue culture conditions such as a 3D culture system or even in vivo animal models to add the dimension of tissue penetration to the current research outcome.

## 4. Materials and Methods

### 4.1. Chemicals and Drugs

Gemcitabine (GCB), terrein (Terr), and sulforhodamine-B were purchased from Sigma Chemical Co. (St. Louis, MO, USA). RPMI-164 media, DMEM, fetal bovine serum, and other cell culture materials were purchased from ATCC (Houston, TX, USA). Other reagents used were of the highest analytical grade.

### 4.2. Cell Culture

Colorectal cancer cell lines HCT-116 (Accession number: CRL-3504), HT-29 (Accession number: HTB-38), and SW620 (Accession number: CVCL_0547) were obtained from ATCC (Houston, TX, USA). Cells were maintained in RPMI-1640 and DMEM supplemented with 100 μg/mL streptomycin, 100 units/mL penicillin, and 10% heat-inactivated fetal bovine serum in a humidified, 5% (*v/v*) CO_2_ atmosphere at 37 °C [67]. All cell lines and cell line materials were confirmed to be mycoplasma free.

### 4.3. Cytotoxicity Assay

The cytotoxicity of GCB and Terr was tested against HCT-116, HT-29, and SW620 cells using the SRB assay as previously described. Exponentially growing cells were harvested using 0.25% Trypsin-EDTA and plated in 96-well plates, at concentrations of 1000–2000 cells/well. Cells were exposed to GCB, Terr, and GCB + Terr for 72 h (normoxia and hypoxia) and subsequently fixed with TCA (10%) for 1 h at 4 °C. After the plates were washed several times, the cells were exposed to a 0.4% SRB solution for 10 min in the dark and subsequently washed with 1% glacial acetic acid. After leaving the plates to dry overnight, Tris-HCl was used to dissolve the SRB-stained cells, and their color intensity was measured at 540 nm using a microplate reader [68].

### 4.4. Data Analysis

The viability dose–response curve of the compounds was analyzed using the Emax model (Equation (1)).

(1) $$\%\;Cell\;viability=\left(100-R\right)\times \left(1-\frac{{\left[D\right]}^{m}}{K_{d}^{m}+{\left[D\right]}^{m}}\right)+R$$

where R is the residual unaffected fraction (the resistance fraction) which is deduced from fitting concentration versus viability on the *E_max_* equation (Equation (1)) described above, [D] is the drug concentration used, *K_d_* is the drug concentration that produces a 50% reduction in the maximum inhibition rate and *m* is a Hill-type coefficient. IC_50_ was defined as the drug concentration required to reduce optical density to 50% of that of the control (i.e., *K_d_* = IC_50_ when R = 0 and *E_max_* = 100 − R) [68].

### 4.5. Apoptosis

Annexin V conjugates allow for the identification of cell surface changes that occur early during the apoptotic process using flow cytometry. Early in the apoptotic process, phosphatidylserine emerges from within the cytoplasmic membrane and becomes exposed on the cell surface, which is thought to be important for macrophage recognition of cells undergoing apoptosis. The binding of Annexin V to phosphatidylserine is calcium-dependent, reversible, and specific with a *K_d_* of approximately 5 × 10 − 10 M [69].

### 4.6. Assessment of Active Caspase-3 Concentration

To assess the effect of GCB, terrein, and their combination on apoptosis, the active caspase-3 concentration was measured using a Quantikine^®^ caspase-3 ELISA Kit (R&D Systems, Inc., Minneapolis, MN, USA). Briefly, the cells were exposed to the predetermined IC_50_s of test compounds (single or combined treatments) or drug-free media (control group) for 24 h. Cells were harvested and washed twice with PBS, then incubated with the biotin-ZVKD-fmk inhibitor for 1 h. Cells were transferred into the wells of a microplate pre-coated with a monoclonal antibody specific for caspase-3. Following a wash to remove any unbound substances, streptavidin conjugated to horseradish peroxidase was added to the wells and bound to the biotin on the inhibitor. Following a wash to remove any unbound streptavidin–HRP, a substrate solution was added to the wells. The enzyme reaction yields a blue product that turned yellow when a stop solution was added. The optical density of each well was determined within 30 min, using a microplate reader set to 450 nm with a wavelength correction at 540 nm or 570 nm. The concentrations of active caspase-3 were calculated from a standard curve constructed with known concentrations of active caspase-3. Caspase concentration was expressed as ng/mg protein. Proteins were determined by the Bradford method using purified bovine serum albumin as a standard protein.

### 4.7. Autophagy

Acridine orange (AO) is a cell-permeable green fluorophore that can become hydronated and consequently absorbed by acidic vesicular organelles. Its metachromatic shift from green to red fluorescence is highly dependent on its concentration, which causes AO to fluoresce from green to red in acidic organelles, such as lysosomes. Lysosomes tend to increase in number and volume when autophagy occurs; AO staining is a quick and reliable method for the assessment of autophagy [70].

### 4.8. Cell Cycle Analysis

Propidium iodide (PI) is a dye that can be used to stain DNA content by intercalating into a double-stranded nucleic acid, producing a highly fluorescent signal when excited at 488 nm with a broad emission centered around 600 nm. The stoichiometric nature of PI ensures accurate quantification of DNA content and reveals the distribution of cells in the G1, S, and G2 cell cycle stages, and even in the sub-G1 cell death stage, which is characterized by DNA fragmentation. Since PI can also bind to double-stranded RNA, it is necessary to treat the cells with RNase for optimal DNA resolution [71].

### 4.9. Gene Expression Analysis

To assess the gene expression of GCB and Terr and their combination, total RNA extraction from cells was performed using the easy-BLUE Kit^®^ (Qiagen Inc., Valencia, CA, USA). Reverse transcription was undertaken to construct a cDNA library from different treatments using a High-Capacity cDNA Reverse Transcription Kit (Applied Biosystems, Foster City, CA, USA). The archived cDNA libraries were then subjected to quantitative real-time PCR reactions [72] using SYBR-green fluorophore (Fermentas Inc., Glen Burnie, MD, USA). Primer sequences were as shown in Table 3.

### 4.10. Metabolomics Analysis

#### 4.10.1. Sample Processing for NMR Spectroscopy

The lyophilized extracellular cell media were mixed with the internal reference 3-(Trimethylsilyl)-1- propane sulfonic acid-d6 sodium salt (DSS-d6, dissolved in methanol-d4, 10 mM) to reach the final concentration of 1 mM. From each sample, 600 μL was placed in 5 mm NMR tubes for NMR analyses. Three biological replicates from each sample were analyzed [73].

#### 4.10.2. NMR Measurement

The ^1^H-NMR experiments were carried out using a Bruker NMR spectrophotometer (Bruker Biospin GmbH, Karlsruhe, Germany) operating at 600 MHz and a temperature of 25 °C.

#### 4.10.3. NMR Spectral Processing

Metabolite annotation was conducted using ChenomX NMR Suite 8.6 (ChenomX Inc., Edmonton, AB, Canada), and phase and baseline corrections were performed initially. The identification was then verified by Human Metabolome Database (http://www.hmdb.ca/) accessed on 1 April 2021, Madison Metabolomics Consortium Database (http://mmcd.nmrfam.wisc.edu) accessed on 1 April 2021. The metabolites with corresponding concentrations were subjected to multivariate analysis [73].

#### 4.10.4. Multivariate Analysis

Metabolite data from all replicates were then imported to MetaboAnalyst 5.0 platform (http://www.metaboanalyst.ca/) accessed on 5 May 2021, for multivariate analysis [74]. Hierarchical cluster analyses were performed to visualize the grouping resulting from the difference between the metabolites released from the HCT-116 cells treated with terrein, GCB, and GCB + Terr under both normoxic and hypoxic conditions. Partial least squares discriminant analysis (PLS-DA) was performed to visualize the grouping tendencies in the samples with many variables. Significant metabolites were determined from variable importance in projection scores (VIP) values. VIP values above 1.00 were significant [75].

### 4.11. Statistical Analysis

Data are presented as mean ± SD. Analysis of variance (ANOVA) with Tukey’s Honest Significance post hoc test was carried out to test for significance using SPSS^®^ for Windows, Version 17.0.0. *p* < 0.05 was used as the cut-off value for significance.

## 5. Conclusions

In conclusion, while combining terrein with gemcitabine did not improve gemcitabine’s resistance fraction, it did however improve its cytotoxic effect against HCT-116 and SW620 cells. The current work focused on HCT-116 as it was the cell line of interest due to the opposite effect of the combination treatment (GCB + Terr) on the cells when treated under normoxic versus hypoxic conditions. Expression of certain genes was affected due to the variable treatment action, more specifically BCL2, Beclin-1, CDK4, and AKT1. This urged us to further investigate the metabolic profile of the HCT-116 cell line after treatment with terrein, gemcitabine, and their combination, and promising results supported our findings. A difference between the metabolites found under each oxygen condition was found, and this could explain the synergistic effect in normoxia versus the antagonistic effect in hypoxia.

## Figures and Tables

**Table 1:** Combination analysis for GCB and Terr against HCT-116, HT-29, and SW620 colorectal cancer cell lines. (N) indicates normoxia, and (H) indicates hypoxia.

	HCT-116		HT-29		SW620	
	IC_50_ (μM)	R-Fraction (%)	IC_50_ (μM)	R-Fraction (%)	IC_50_ (μM)	R-Fraction (%)
GCB (N)	0.19 ± 0.028	38.14 ± 1.40	0.01 ± 0.006	19.87 ± 12.0	0.21 ± 0.0003	32.61 ± 1.78
Terr (N)	75.22 ± 0.97	N/A	56.24 ± 11.39	N/A	72.28 ± 1.35	8.34 ± 1.37
GCB + Terr (N)	0.023 ± 0.005	24.40 ± 1.22	0.027 ± 0.005	46.45 ± 2.76	0.018 ± 0.04	32.30 ± 3.56
CI value	0.13		2.32		0.09	
GCB (H)	0.01 ± 0.002	0.0	0.04 ± 0.004	42.64 ± 2.10	0.20 ± 0.008	32.59 ± 0.28
Terr (H)	20.26 ± 2.89	8.13 ± 0.98	83.30 ± 4.41	3.02 ± 3.39	59.82 ± 8.50	2.52 ± 3.39
GCB + Terr (H)	0.024 ± 0.03	41.02 ± 2.33	0.324 ± 7.95	66.74 ± 6.84	0.027 ± 0.007	48.51 ± 2.97
CI value	1.78		7.28		0.14	

**Table 2:** Extracellular metabolites identified by ^1^H NMR-based profiling for HCT-116 cells under both hypoxic and normoxic conditions. The quantification of metabolites was achieved via fitting with its reference spectrum from the library of the Chenomx NMR suite. The mean concentration ± standard error is shown for metabolites from a set of three biological replicates.

Name	NMR Chemical Shift	Concentration (mM)							
		Normoxia				Hypoxia			
		Control	Terr	GCB	Terr + GCB	Control	Terr	GCB	Terr + GCB
4-hydroxyphenyl acetate	7.16 (d), 6.68 (t), 3.44 (s)	0	3.2866	2.342	2.4149	0	0	0	1.332
2-hydroxyvalerate		0	0	0	0	0	7.351	26.6519	7.033
2-phosphoglycerate	4.86 (dt), 3.76 (dd), 3.62 (dd)	0	0	0	0	0	92.55	165.354	10.4
Acetate	1.9 (s)	0	4.203	6.2544	2.8006	0	3.2218	6.9808	2.04
Alanine	1.46 (d)	8.7211	18.9587	20.264	16.0552	48.545	25.8158	19.2287	1.1259
Dimethylamine	2.5 (s)	0	20.6557	6.4366	14.3796	0	18.5201	4.2958	13.026
Ethylmalonate		0	0	11.0424	0	0	10.0587	9.8134	4.29
Formate	8.46 (s)	1.3573	3.5965	4.6718	0	0	2.0378	4.6042	1.2927
Glucose	3.23, 3.39, 3.45, 3.50, 3.71, 3.81, 3.88, 4.63, 5.22	71.7887	143.9592	193.9061	102.7318	257.7746	71.1131	108.3805	36.567
Glutamate	2.34 (m)	7.9295	17.862	8.3554	31.9252	31.8823	5.9122	29.2106	7.0509
Histamine	7.99 (s), 7.14 (s), 3.29 (t), 3.03 (m)	0	2.2367	2.0502	2.993	0	1.2942	1.0424	1.9017
Hypoxanthine	8.19 (s), 8.21 (s)	0	1.1172	0	1.3887	0	0.7355	0	1.53
Imidazole	7.28 (s), 8.18 (s)	0	3.6693	0	1.4086	0	0.8819	0	0.99
Lactate	1.31, 4.09	62.6687	144.9777	201.7872	73.772	400.2995	103.39	166.539	65.067
Leucine	0.96 (t),1.70 (m)	3.7907	8.944	9.7745	4.7783	0	0	0	3.048
Methionine	3.86 (dd), 2.65 (t), 2.23 (m)	0	6.5637	0	0	0	0.4214	0	0.84
Phenylalanine	7.37 (m), 3.98 (m), 3.27 (m), 3.11 (m)	1.4766	3.7121	1.9949	3.3628	4.721	1.3442	0	1.1259
Pyruvate	2.36 (s)	0	0	0	0	0	0	0	4.7677
Pipecolate	3.58 (dd), 3.42 (m), 3.02 (td), 2.22 (m), 1.89 (m), 1.69 (m)	0	31.4662	12.192	0	0	0	0	0
Succinate	2.39 (s)	1.4136	2.4953	0.6909	2.3995	6.888	0.4962	0.1574	0.1986
Tyrosine	7.17 (m), 6.8 (m), 3.9 (m)	1.0549	0	2.8125	0	5.7714	1.2658	1.7629	1.28
Tyramine	2.92 (t), 3.23 (t), 6.9 (m), 7.2 (m)	0	3.4448	2.7521	2.6895	0	0	0	0
Valine	3.6 (d), 2.29 (m), 1.04 (d), 0.98 (d)	2.1199	3.6865	5.2247	2.535	7.3598	2.204	2.6834	2.037
Xanthine	7.89 (s)	0	1.7549	1.6411	1.6685	0	0.9064	0	0.6265
Methylhistidine	7.67 (s), 7.0 (s), 3.97 (dd), 3.68 (s), 3.18 (dd), 3.09 (dd)	0.5439	0	1.535	0	0	0	0	0

**Table 3:** Primer sequences of target genes were used for the qPCR analysis.

Classification	Primer	Direction	Code
Apoptosis regulators	BCL2	Forward	GAT-TGT-GGC-CTT-CTT-TGA-G
		Reverse	CAA-ACT-GAG-CAG-AGT-CTT-C
	BIRC5	Forward	AGG-ACC-ACC-GCA-TCT-CTA-CAT
		Reverse	AAG-TCT-GGC-TCG-TTC-TCA-GTG
	TP53	Forward	TTC-CTC-CAA-CCA-AGA-ACC-AGA
		Reverse	GCT-CAG-TAG-GTG-ACT-CTT-CAC-T
	FOXO3	Forward	ACG-GCT-GAC-TGA-TAT-GGC-AG
		Reverse	CGT-GAT-GTT-ATC-CAG-CAG-GTC
Autophagy	ATG5	Forward	AGA-AGC-TGT-TTC-GTC-CTG-TGG
		Reverse	AGG-TGT-TTC-CAA-CAT-TGG-CTC
	Beclin1	Forward	AGC-TGC-CGT-TAT-ACT-GTT-CTG
		Reverse	ACT-GCC-TCC-TGT-GTC-TTC-AAT-CTT
Cell cycle regulators	CCND1	Forward	TGT-TCG-TGG-CCT-CTA-AGA-TGA-AG
		Reverse	AGG-TTC-CAC-TTG-AGC-TTG-TTC-AC
	CDK4	Forward	CTG-GTG-TTT-GAG-CAT-GTA-GAC-C
		Reverse	AAA-CTG-GCG-CAT-CAG-ATC-CTT
	MCM7	Forward	GGG-CTC-CAG-ATT-CAT-CAA-AT
		Reverse	ATA-CCA-GTG-ACG-CTG-ACG-TG
Cell growth	AKT1	Forward	GGA-TGT-GGA-CCA-ACG-TGA-G
		Reverse	AGC-GGA-TGA-TGA-AGG-TGT-TG
	TGF-β1	Forward	GGT-ACC-TGA-ACC-CGT-GTT-GCT
		Reverse	TGT-TGC-TGT-ATT-TCT-GGT-ACA-GCT-C
	HIF1-a	Forward	GAA-CGT-CGA-AAA-GAA-AAG-TCT-CG
		Reverse	CCT-TAT-CAA-GAT-GCG-AAC-TCA-CA
	PRKDC	Forward	GAC-ATC-TCC-TGA-GCT-CTG-AC
		Reverse	CTC-TTG-TTC-CCC-AAC-AGT-CT
Cell proliferation	PCNA	Forward	CGG-ATA-CCT-TGG-CGC-TAG-TA
		Reverse	TCT-CGG-CAT-ATA-CGT-GCA-AA
	RAD18	Forward	CTC-AGT-GTC-CAA-CTT-GCT-GTG
		Reverse	GAA-GAG-GAA-GAA-GCA-GGA-GAT

## Data Availability

All supporting raw data are available upon request.

## Supplementary Materials

The following supporting information can be downloaded at: https://www.mdpi.com/article/10.3390/md21050271/s1, Figure S1: The effect of terrein on the cytotoxicity of GCB (A) in normoxia and (B) in hypoxia in HCT-116 cell line. The cells were exposed to serial dilution of terrein (○), GCB (•) or Terr/GCB combination (▾) for 72 h. The cell viability was determined using SRB assay; Figure S2: The effect of terrein on the cytotoxicity of GCB (A) in normoxia and (B) in hypoxia in HT-29 cell line. The cells were exposed to serial dilution of terrein (○), GCB (•) or Terr/GCB combination (▾) for 72 h. The cell viability was determined using SRB assay; Figure S3: The effect of terrein on the cytotoxicity of GCB (A) in normoxia and (B) in hypoxia in SW620 cell line. The cells were exposed to serial dilution of terrein (○), GCB (•) or Terr/GCB combination (▾) for 72 h. The cell viability was determined using SRB assay.
