# Supplementary material for: Chemomodulatory Effect of the Marine-Derived Metabolite “Terrein” on the Anticancer Properties of Gemcitabine in Colorectal Cancer Cells
Source: Mar Drugs. 2023 Apr 26;21(5):271. doi: 10.3390/md21050271 (PMC10221861; doi:10.3390/md21050271)
Supplement: Supplementary file 1 [file marinedrugs-21-00271-s001.zip › marinedrugs-2335180-supplementary.pdf]

(**A**) (**B**)

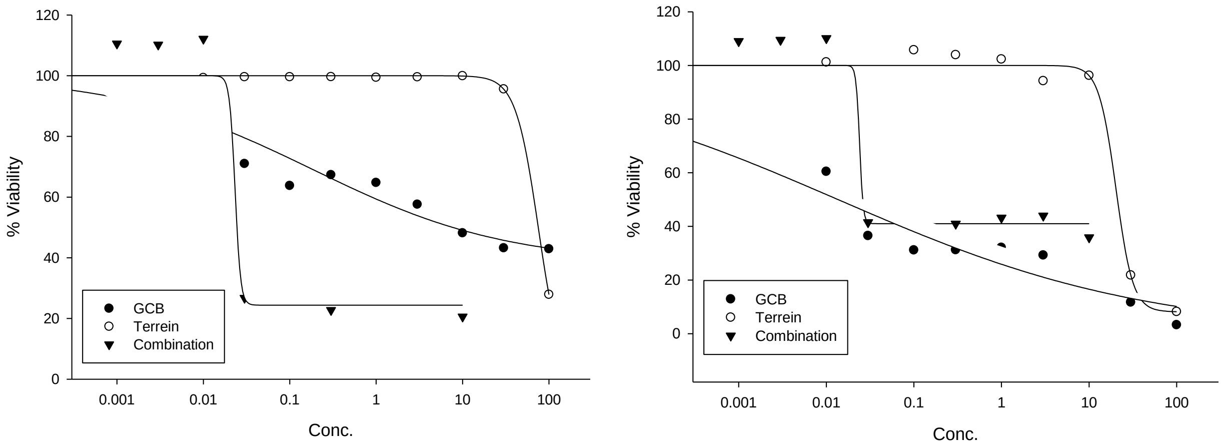

Supp. Figure S1. The effect of terrein on the cytotoxicity of GCB (A) in Normoxia and (B) in hypoxia in HCT-116 cell line. The cells were exposed to serial dilution of terrein (○), GCB (•) or Terr/GCB combination (▾) for 72 h. The cell viability was determined using SRB assay.

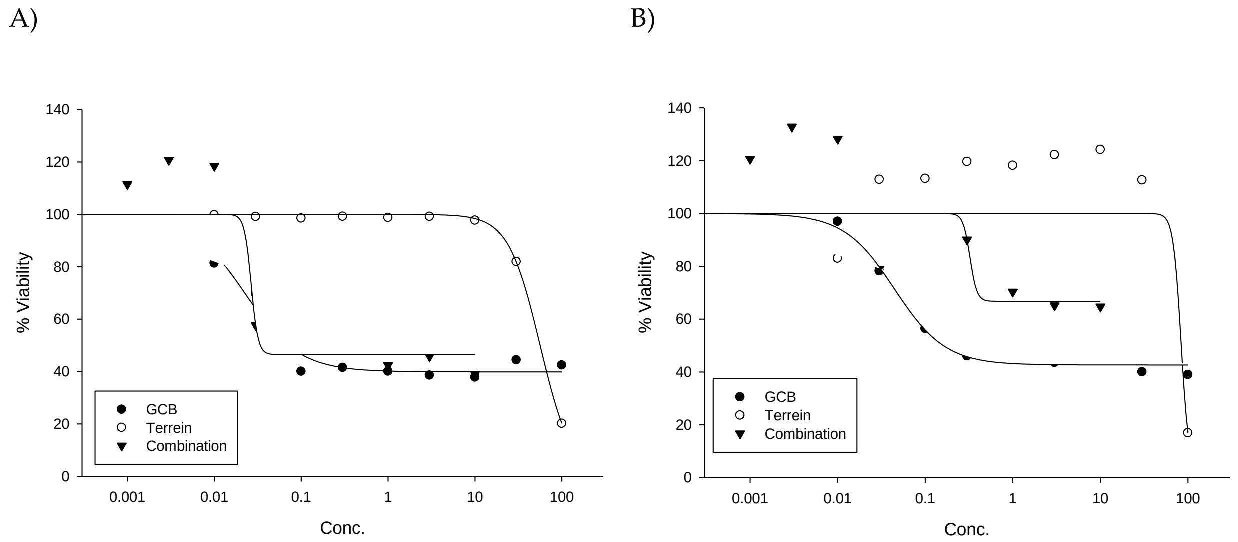

Supp. Figure S2. The effect of terrein on the cytotoxicity of GCB (A) in Normoxia and (B) in hypoxia in HT-29 cell line. The cells were exposed to serial dilution of terrein (○), GCB (•) or Terr/GCB combination (▾) for 72 h. The cell viability was determined using SRB assay.

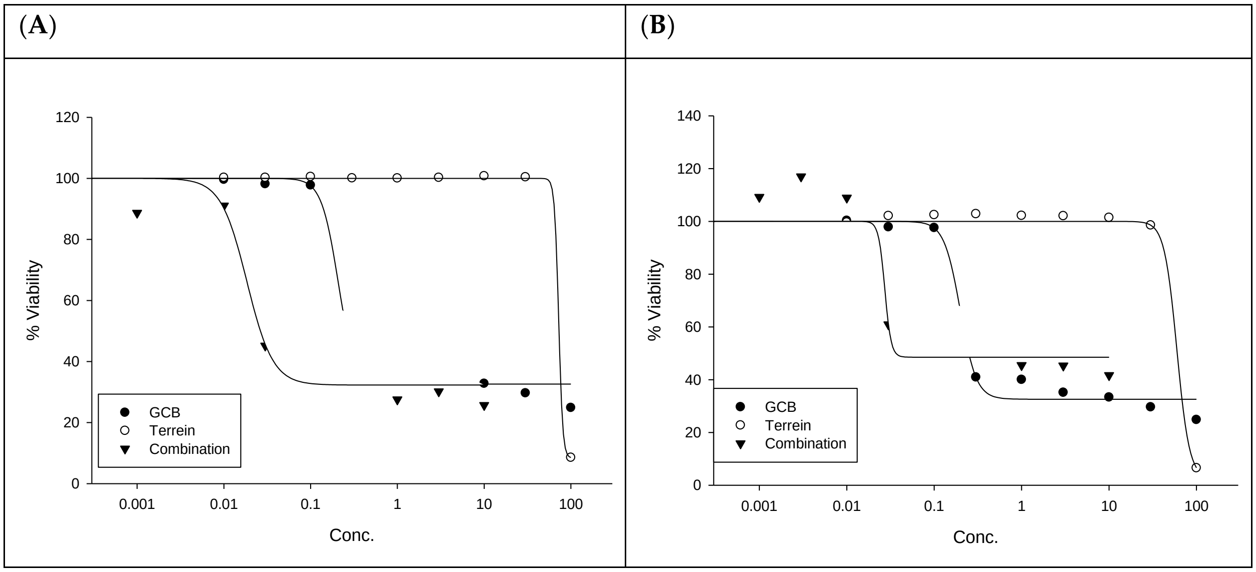

Supp. Figure S3. The effect of terrein on the cytotoxicity of GCB (A) in Normoxia and (B) in hypoxia in SW620 cell line. The cells were exposed to serial dilution of terrein (○), GCB (•) or Terr/GCB combination (▾) for 72 h. The cell viability was determined using SRB assay.
